# Reactive Oxygen Species Is Essential for Cycloheximide to Sensitize Lexatumumab-Induced Apoptosis in Hepatocellular Carcinoma Cells

**DOI:** 10.1371/journal.pone.0016966

**Published:** 2011-02-10

**Authors:** Xiangxuan Zhao, Mengde Cao, Joy J. Liu, Haizhen Zhu, David R. Nelson, Chen Liu

**Affiliations:** 1 Departments of Pathology, Immunology, and Laboratory Medicine, University of Florida College of Medicine, Gainesville, Florida, United States of America; 2 Department of Medicine, University of Florida, Gainesville, Florida, United States of America; Vanderbilt University Medical Center, United States of America

## Abstract

This study aims to investigate apoptosis induced by lexatumumab (Lexa) in hepatocellular carcinoma (HCC) cells. We assessed the sensitivity of HCC cell lines and normal human hepatocytes to Lexa and explored the sensitization of HCC cells to Lexa-induced apoptosis by cycloheximide (CHX). Our data indicated that CHX sensitized HCC cell lines to Lexa-induced apoptosis, whereas treatment using solely CHX or Lexa was ineffective. The sequential treatment of CHX followed by Lexa dramatically induced caspase-dependent apoptosis in HCC cells and had synergistically increased intracellular rates of reactive oxygen species (ROS). Additionally, when ROS production was blocked by N-acetyl-L-cysteine (NAC), HCC cells were protected against Lexa and CHX combination treatment-induced apoptosis. ROS generation induced by combination treatment of Lexa and CHX triggered pro-apoptotic protein Bax oligomerization, conformation change, and translocation to mitochondria, which resulted in the release of cytochrome c and subsequent cell death. Furthermore, HSP90 was involved in mediating Lexa and CHX combination treatment-induced ROS increase and apoptotic death. More importantly, we observed that combination treatment of Lexa and CHX did not cause apoptotic toxicity in normal human primary hepatocytes. These results suggest that Lexa and CHX combination treatment merits investigation for the development of therapies for patients with HCC.

## Introduction

Hepatocellular cancer is one of the five most common cancers worldwide and is fatal in more than 90% of patients [1]. Currently, there are no effective therapies for liver cancer other than surgical resection or liver transplantation in the early stages of tumor development. Such treatments only apply to a small percentage of patients, while the majority die within 6 months of diagnosis [2]. Therefore, new therapeutic strategies are urgently needed.

Targeting death receptor activation-mediated cell death is quickly becoming one of the most promising strategies for anti-cancer therapy [3], [4]. An overwhelming number of studies have demonstrated that the administration of death receptor agonists can selectively induce apoptosis in tumor cells and significantly inhibit xenograft human tumor growth [5]–[8]. Among the death receptor agonists, lexatumumab (Lexa) was developed as a potential humanized anti-death receptor 5 (DR5) monoclonal antibody. It has been shown that Lexa specifically binds to death receptor 5 and induces apoptosis in a number of tumor cell lines, including renal cell carcinoma (RCC) [9], human myeloma cell lines (HMCL) [10], and malignant pleural mesothelioma (MPM) [11]. Different researchers have also reported that combination treatment with agonistic death receptor 5 mAbs and chemotherapeutic drugs exert a synergistic apoptotic effect in some tumor cell lines, such as lymphoma [12]–[14], breast cancer [15], colorectal cancer [16], and malignant mesothelioma [11]. Nevertheless, it remains unknown whether Lexa can induce apoptosis in hepatocellular carcinoma (HCC) cells or whether it has apoptotic toxicity to normal hepatocytes.

In the present study, we are the first to show data indicating that Lexa can significantly induce apoptosis in resistant HCC cell lines in the presence of cycloheximide (CHX). We provide evidence to demonstrate that treatment combining Lexa and CHX induces caspase-dependent apoptosis in HCC cells. Intracellular reactive oxygen species (ROS) generation, Bax/Bak activation, and heat shock protein 90 (HSP90) inactivation are involved in killing the HCC cells. More importantly, we found that Lexa and CHX combination treatment has no apparent apoptotic toxicity to normal human hepatocytes.

## Materials and Methods

### Cell culture and reagents

Human hepatocellular carcinoma cell lines, Huh7 and LH86 were grown in Dulbecco's Modified Eagle's Medium (DMEM) with 10% fetal bovine serum (Sigma, St. Louis, MO) and antibiotics (100 U/ml penicillin and 100 µg/ml streptomycin) at 37°C in 5% CO_2_. Normal primary human hepatocytes were obtained from CellzDirect Inc (Austin, TX). The cells were cultured in DMEM/F12 (1∶1) culture medium. The human normal hepatocytes used were at least 90% viable before treatment. Anti-caspase 8, anti-caspase 10, anti-caspase 3, anti-cytochrome c, anti-HSP90, anti-Bcl-xL, anti-IKK-β, anti-IκB-α, anti-p-IκB-α, anti-Mcl-1, anti-Bak, anti-DR4, anti-DR5, and anti-Bid primary antibodies were obtained from Cell Signaling Technology(Beverly, MA); Dihydroethidium (DHED), N-acetyl-L-cysteine (NAC), Bis (maleimido) hexane (BMH)/DSS, DMAG-17, Mito Tracker (Red) CMXRos, IKK inhibitor, NEMO-binding domain peptide (NBD): MAPK inhibitor: PD98059, P38 inhibitor: SB203580, and JNK inhibitor: SP600125 were obtained from Invitrogen (Carlsbad, CA); anti-β-actin, anti-Bax 6A7 monoclonal antibodies, Hoechst 33258, and 4′, 6′-Diamidino-2-phenylindole (DAPI) were obtained from Sigma (St. Louis, MO); z-IETD-FMK and z-VAD-FMK were obtained from Calbiochem (San Diego, CA). Anti-Bax (N-20) primary polyclonal antibody, goat anti-rabbit horseradish peroxidase (HRP) conjugated secondary antibody, Goat anti-rabbit secondary antibody conjugated with FITC, and protein G plus-agarose were purchased from Santa Cruz Biotechnology (Santa Cruz, CA). The Annexin-V apoptosis detection kit was obtained from BD Bioscience (San Diego, CA). Lexatumumab was kindly provided by Human Genome Science Inc. [12], [17].

### Hoechst staining assay

Apoptosis was determined through nuclear morphology change. After treatment with different stimuli, cells were stained with Hoechst 33258 at 37°C for 10 min. Cellular DNA fragmentation/nucleus condensation was detected using an Olympus fluorescent microscope. Apoptotic cell death ratio was assessed through calculating the number of apoptotic cells with condensed nuclei in six to eight randomly selected areas.

### DNA ladder assay

A DNA ladder assay was performed as previously described [18] with modification. Briefly, cells treated under different conditions were harvested and washed with 1×phosphate buffered saline (PBS). Cell pellets were resuspended with lysis buffer (1% NP-40 in 20 mM EDTA, 50 mM Tris-HCl, pH 7.5). The samples were centrifuged at 1,600×*g* for 5 min and supernatants were transferred to new tubes. SDS (final concentration 1%) and RNase A (final concentration 5 µg/ml) were added to the supernatants, and the mixture was incubated at 56°C for 2 h. After addition of proteinase K (final concentration 2.5 µg/ml), the samples were incubated at 37°C for 2 h. DNA was precipitated with the addition of 10 M ammonium acetate and ethanol, washed once with 70% ethanol and then dissolved in water and separated by electrophoresis in 1% agarose gel.

### Fluorescence assisted cell sorting (FACS) assay

Apoptosis was measured using the Annexin-V detection kit according to the manufacturer's instructions. Flow cytometric analysis was performed to monitor the green fluorescence of the FITC-conjugated Annexin-V and the red fluorescence of DNA-bound propidium iodide (PI). All data were analyzed with a Cell Quest software (BD).

### Western blotting analysis

Cells were harvested and washed twice with 1×PBS. The cell pellets were resuspended in lysis buffer containing Nonidet P-40 (10 mM HEPES, pH 7.4, 2 mM EGTA, 0.5% Nonidet P-40, 1 mM NaF, 1 mM NaVO_4_, 1 mM phenylmethylsulfonyl fluoride, 1 mM dithiothreitol, 50 µg/ml trypsin inhibitor, 10 µg/ml aprotinin, and leupeptin) and incubated on ice for 30 min. After centrifugation at 12,000×*g* at 4°C for 15 min, the supernatant was transferred to a new tube and the protein concentration was determined. Equivalent samples (20 µg of protein) were subjected to SDS-PAGE on 12% gels. The proteins were transferred to nitrocellulose membranes and probed with the indicated primary antibodies, followed by the appropriate secondary antibodies conjugated with horseradish peroxidase. Immunoreactive bands were detected using enhanced chemiluminescence (ECL) (Pierce, Rockford, IL). The molecular sizes of the proteins detected were determined by comparison with prestained protein markers (Bio-Rad, Hercules, CA).

### Immuno-fluoresence staining

Immunostaining was performed as described previously [19] with modification. Briefly, cells seeded on cover-slips in culture dishes were treated with Lexa in the presence of CHX for 6 h. Mito-Tracker (Red) was directly added to cultures and incubated for 15 min. Cells were washed with 1× PBS, fixed with 3.7% paraformaldehyde (PFA) at 37°C for 15 min, and then quenched with 50 mM NH_4_Cl. After washing with PBS twice, cells were permeabilized with 0.2% Triton X-100 in PBS, then blocked with PBS containing 5% goat serum for 30 min. Cells were further incubated with primary antibody cytochrome c overnight at 4°C, followed by staining with FITC conjugated goat anti-rabbit IgG (1∶200) for 2 h at room temperature. The cover-slips were then extensively washed and mounted in Mowiol with DAPI dye. Fluorescent images were obtained using an Olympus fluorescent microscope.

### Bax cross-linking

In vitro protein cross-linking was carried out as previously described [20] with minor modification. Cells treated with different conditions were harvested. Cell lysates were prepared after lysis with HEPES-CHAPS lysis buffer (20 mM HEPES pH 7.2, 150 mM NaCl, 2 mM EDTA, 10 mM glucose, 2% CHAPS). Bis (maleimido) hexane (BMH) substrate or dimethyl sulfoxide (DMSO) was added at a final concentration of 2 mM. The samples were incubated at room temperature for 30 min and quenched by the addition of 1 M Tris-HCl (pH 7.5). An in vivo protein cross linking assay was performed as previously described. Following treatments with Lexa or Lexa combined with CHX, cells were washed with conjugating buffer containing 150 µM NaCl, 20 mM HEPES (pH 7.2), 1.5 mM MgCl_2_, and 10 mM glucose. DSS in DMSO was added for a final concentration of 2 mM. After reaction at room temperature for 30 min, the cross-linker was quenched by the addition of 1 M Tris-HCl (pH 7.5) for a final concentration of 20 mM. Samples were then solubilized in 1% NP-40 and centrifuged at 12,000×*g* for 10 min. A 20-µl aliquot from the 400 µl cross-linked lysate was analyzed by Western blotting with Bax (N-20) polyclonal antibody.

### ROS detection

The intracellular production of ROS was measured by fluorogenic probe dihydroethidium-(DHED) based fluorescent staining. Briefly, after various treatments, cells were incubated with DHED (5 µM) for 30 min at 37°C, then rapidly rinsed twice with 1× PBS and observed under fluorescent microscope.

### siRNA knockdown

siRNA knockdown was performed as previously described [21]. Smart-pool pre-designed siRNA duplexes targeted against human HSP90 mRNA were from Cell Signaling Technology (Beverly, MA). HCC cells were plated at a density of 1×10^6^ cells/well in 6-well plates (BD Bioscience, San Diego, CA). Next day, cells were transfected with 100 nM siRNA duplex mixtures for 24 h in the presence of lipo-fectamine RNAiMax (Invitrogen, Carlsbad, CA) according to the manufacturer's instructions. A non-specific random siRNA (Cell Signaling Biotechnology, Beverly, MA) was also transfected at the same concentration as control.

### Subcellular fractionation

This assay was performed as described previously [22]. Cells were harvested and resuspended in three volumes of hypotonic buffer (210 mM sucrose, 70 mM mannitol, 10 mM HEPES (pH 7.4), 1 mM EDTA) containing 1 mM phenylmethylsulfonyl fluoride (PMSF), 50 µg/ml trypsin inhibitor, 10 µg/ml leupeptin, 5 µg/ml aprotinin, and 10 µg/ml pepstatin. After gentle homogenization with a Dounce homogenizer, cell lysates were centrifuged at 1000×*g* for 5 min to remove unbroken cells and nuclei. The postnuclear supernatant was centrifuged at 10,000×*g* to pellet the mitochondria-enriched heavy membrane fraction. The supernatant was further centrifuged at 100,000×*g* to obtain the cytosolic fraction. A 30-µl aliquot from the 500 µl cytosolic fraction was analyzed by Western blotting with anti-cytochrome c monoclonal antibody.

### Detection of Bax conformation change

This assays was performed as described previously [23]. Cells were lysed with Chaps lysis buffer (10 mM HEPES (pH 7.4), 150 mM NaCl, and 1% Chaps) containing 1 mM phenylmethylsulfonyl fluoride (PMSF), 50 µg/ml trypsin inhibitor, 10 µg/ml leupeptin, 1 µg/ml aprotinin, and 5 µg/ml pepstatin. The cell lysates were normalized for protein content and 500 µg of total protein was incubated with 2 µg of anti-Bax 6A7 monoclonal antibody in 500 µl of Chaps lysis buffer at 4°C for 4 h. Then, 40 µl of protein G plus-agarose were added into the reactions and incubated at 4°C for an additional 2 h. Following three washings in Chaps lysis buffer, beads were boiled in loading buffer, and the conformationally changed Bax protein in the immunoprecipitates was subjected to SDS–PAGE (15% gel) and immunoblot analysis with anti-Bax polyclonal antibody as described above.

## Results

### Lexa induces apoptosis in HCC cells in the presence of CHX

**Figure 1:**
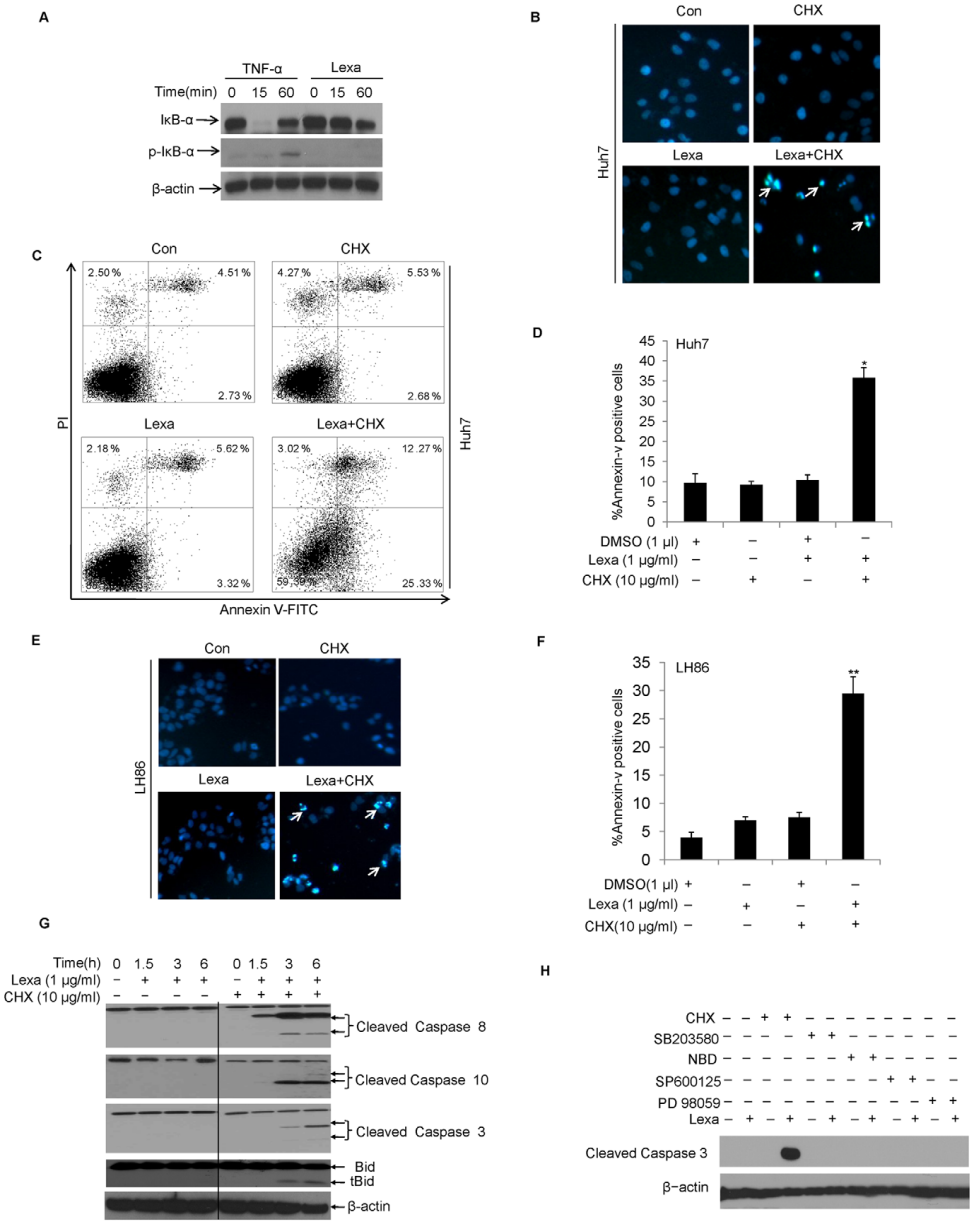
Lexa and CHX combination treatment induces apoptosis in HCC cells. **A,** Huh7 cells were treated with TNF-α (10 ng/ml) or Lexa (1 µg/ml) for indicated times. Cell lysates were prepared and subjected to Western blotting to detect the expression of IκB-α and phospho-IκB-α. β-actin protein levels were used as an equal protein loading control (Lexa, lexatumumab). **B**, Huh7 cells were treated with DMSO (Con), CHX (10 µg/ml), Lexa (1 µg/ml), or a combination of Lexa (1 µg/ml) and CHX (10 µg/ml). Apoptosis was measured by nuclear dye Hoechst 33258 staining to label DNA fragmentation (nuclear morphology changes). (Apoptotic cells were marked with arrows). **C**, Huh7 cells were treated with DMSO (Con), CHX (10 µg/ml), Lexa (1 µg/ml), or combination treatment of Lexa (1 µg/ml) and CHX (10 µg/ml) and apoptosis was evaluated through Annexin V and PI double staining based FACS analysis using the Annexin-V assay kit (see 'materials and methods'). **D**, The percentage of apoptotic cells were characterized as those that stained with Annexin-V. Data represent the mean values of three independent experiments (*p<0.05). **E**, LH86 cells were treated with DMSO (Con), CHX (10 µg/ml), Lexa (1 µg/ml), or a combination of Lexa (1 µg/ml) and CHX (10 µg/ml). Apoptosis was measured as in Fig. 1B (Apoptotic cells were marked with arrows). **F**, LH86 cells were treated with various stimuli as in Fig. 1E. Apoptosis was measured through FACS analysis as in Fig. 1C and 1D and statistical analysis was performed to show percentage of Annexin-V positive cells for apoptosis ratio. Data represent the mean values of three independent experiments (**p<0.05). **G**, Huh7 cells were treated with Lexa (1 µg/ml) or combination of Lexa (1 µg/ml) and CHX (10 µg/ml) for up to 6 h and harvested. Cell lysates were prepared for Western blotting to detect the cleavages of caspase 8, caspase 10, caspase 3, and Bid. β-actin protein levels were set up as loading control for equal total protein amounts. **H**, Huh7 cells treated with indicated conditions for 6 h were harvested and cell lysates were prepared and subjected to Western blotting analysis. Caspase 3 activation was evaluated through detecting cleaved bands with specific antibody. β-actin protein levels were set up as loading control for equal total protein amounts. Each experiment was conducted in triplicate or duplicate and repeated twice independently.

Increasing evidence indicated that many cancer types including breast cancer cells, lung cancer cells, and hepatoma cells, could develop resistance to TRAIL-induced apoptosis through DR5-mediated activation of NF-κB signal pathway [24]–[26]. Here, to investigate whether Lexa triggers NF-κB activation in HCC cell lines, Huh7 cells were treated with TNF-α or Lexa for the indicated times. Western blotting results indicated that TNF-α induced Iκ-B-α decrease after treatment for 15 min and Lexa did that with treatment for 60 min. However, compared with TNF-α, Lexa could not promote Iκ-B-α phosphorylation at 60 min (Fig. 1A); thus Lexa, a DR5 specific monoclonal antibody unlike multifunctional TNF-α, did not activate the anti-apoptotic NF-κB signal pathway through binding to DR5. Then, we tested whether HCC cells are sensitive to Lexa-induced cell death. Huh7 and LH86 cells were treated with Lexa (1 µg/ml) for up to 6 h. Apoptosis assays indicated that both HCC cell lines were resistant to Lexa (see Lexa-treated groups in Fig. 1B, 1C, and 1D for Huh7 cells, and Fig. 1E and 1F for LH86 cells).

It has been shown that chemotherapeutic reagent CHX, a protein synthesis inhibitor, can enhance TRAIL-induced apoptosis in tumor cells such as renal cancer [27], colon cancer [28], and lung carcinoma [29]. As cells did not undergo apoptosis with Lexa single treatment, we examined whether CHX can alter the insensitivity of HCC cells to Lexa. Apoptosis assays indicated that pre-treatment with CHX (10 µg/ml for 30 min) followed by Lexa (1 µg/ml for 6 h) could significantly enhance apoptosis induction in both Huh7 and LH86 cells (Lexa and CHX combination treated groups in Fig. 1B, 1C, and 1D for Huh7 cells, and Fig. 1E and 1F for LH86 cells). To further confirm Lexa-induced apoptosis could be enhanced by CHX pre-treatment, we evaluated caspase activation and the cleavage of the pro-apoptotic protein Bid through Western blotting analysis. Our data indicated that co-treatment with Lexa and CHX for 6 h significantly induced proteolytic processing of caspase 8, 10, and 3, as well as Bid (Fig. 1G, right panel), but single treatment with either Lexa or CHX could not result in cleaved bands or active subunits from procaspase 8, procaspase 10, or procaspase 3. Additionally, Bid cleavage was not progressively processed after 3 h (Fig. 1G, left panel), supporting the assumption that these liver cancer cells are resistant to Lexa-induced apoptosis. To investigate the possible signaling pathways underlying the resistance of HCC cells to Lexa, we used the specific inhibitors NBD, PD98059, SB203580, and SP600125 to respectively inhibit the IKK, MAPK, P38, and JNK signaling pathways. As shown in Fig. 1H, in comparison with positive control that CHX and Lexa combination treatment-induced caspase 3 cleavages, the other four inhibitors did not reverse the insensitivity of HCC cells to Lexa-induced apoptosis. These results suggest that Lexa and CHX combination treatment synergistically induces apoptosis in HCC cells.

### Lexa and CHX combination treatment induces caspase dependent apoptosis

**Figure 2:**
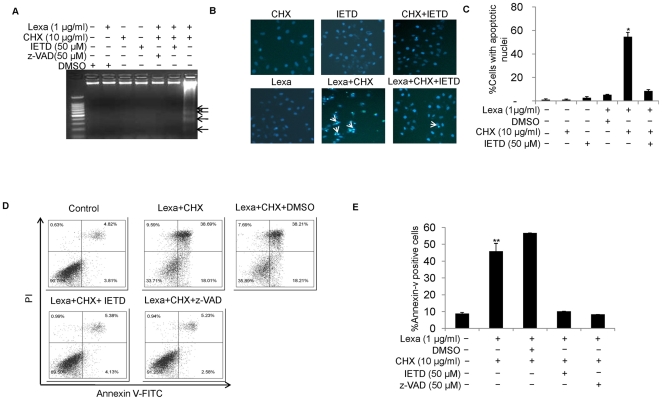
Lexa and CHX combination treatment induces caspase-dependent apoptosis. **A**, Huh7 cells were treated with different conditions as indicated. Apoptosis was tested by DNA ladder assays as described in ‘Materials and Methods’. **B**, Huh7 cells were treated with various stimuli as indicated (Lexa, (1 µg/ml), CHX, (10 µg/ml), or caspase 8 inhibitor IETD (50 µM)). Apoptotic cells were examined as described in Fig. 1B (representative apoptotic cells were marked with arrows). **C**, Huh7 cells were treated with conditions as indicated and apoptotic ratio was determined by counting cells with apoptotic nuclei as described in ‘Materials and Methods’. Data represent the mean values of three independent experiments (*p<0.05). **D**, Huh7 cells were treated with various conditions as indicated (Lexa, (1 µg/ml), CHX, (10 µg/ml), IETD (50 µM), z-VAD (50 µM)). Apoptosis was evaluated as in Fig. 1C. **E**, Huh7 cells were treated with different conditions as indicated and apoptosis percentage was determined as in Fig. 1D. Data represent the mean values of three independent experiments (**p<0.05).

Death receptor-mediated apoptosis can be classified as either caspase dependent or caspase independent [30], [31]. To examine whether Lexa and CHX combination-induced apoptosis is controlled by caspase activation, Huh7 cells were pre-incubated with pan-caspase inhibitor z-VAD-FMK (50 µM) for 1 h, followed by various stimuli as indicated. DNA ladder assays (Fig. 2A), DNA fragmentation analysis (Fig. 2B and 2C) and FACS analysis (Fig. 2D and 2E) demonstrated that apoptosis induced by combination treatment with Lexa and CHX were totally blocked by pan-caspase inhibitor z-VAD-FMK, suggesting that CHX-sensitized and Lexa-induced apoptosis is caspase dependent. Meanwhile, we measured the effects of caspase 8 specific inhibitor z-IETD-FMK on apoptosis induced by Lexa and CHX combination treatment. As shown in Fig. 2A, 2B, 2C, 2D, and 2E, blockade of caspase 8 activation completely abolished Lexa and CHX combination-induced apoptosis in Huh7 cells. Taken together, these results suggest that caspase activation plays a key role in Lexa-mediated apoptosis induction in HCC cells.

### Lexa and CHX combination treatment-induced apoptosis is regulated by ROS

**Figure 3:**
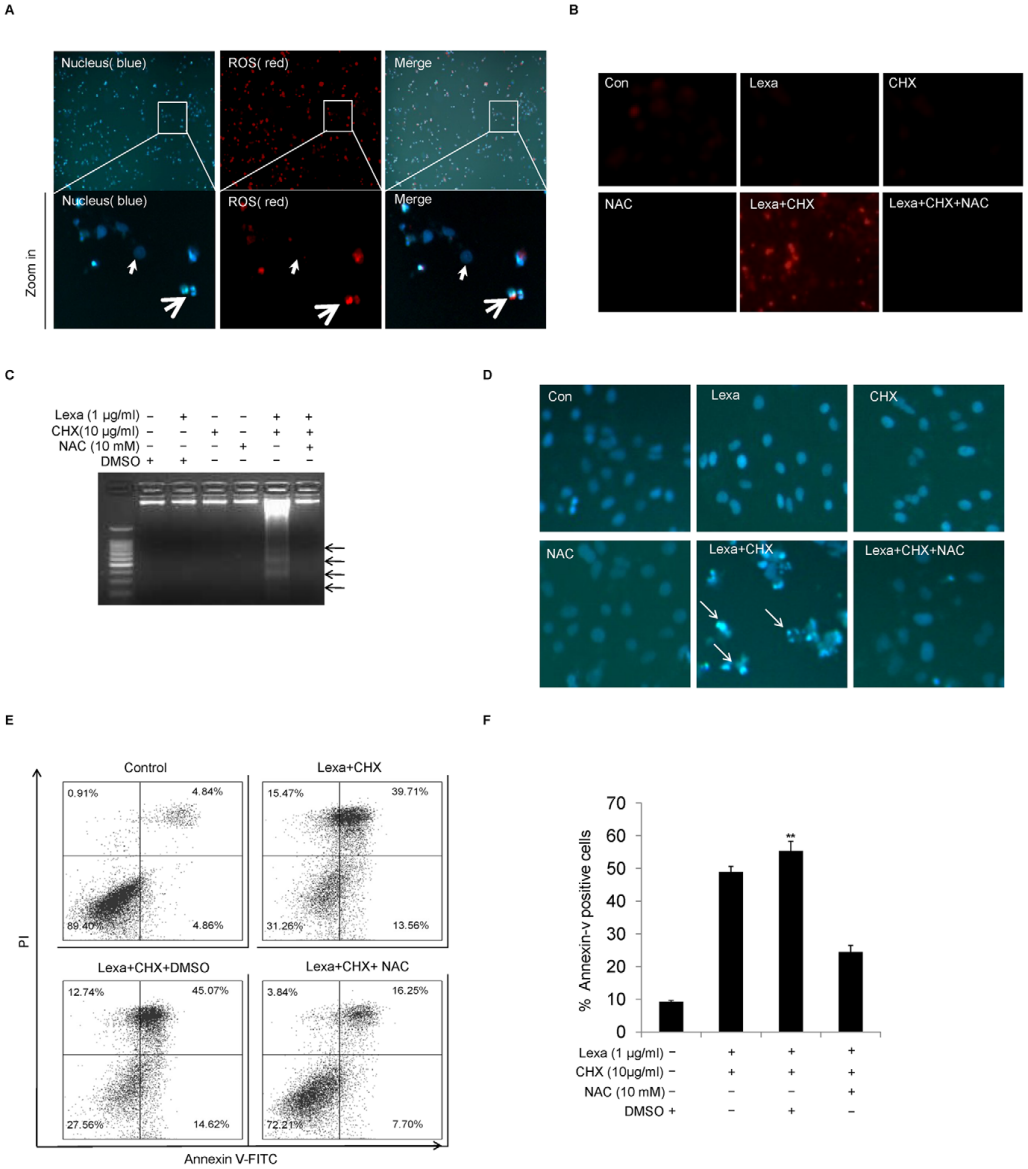
Lexa and CHX combination treatment induces apoptosis via ROS. **A**, Huh7 cells were treated with the combination of Lexa (1 µg/ml) and CHX (10 µg/ml). Nuclei were stained by Hoechst 33258 (right panel-blue, thin arrow marks the nucleus in non-apoptotic cells; thick arrow marks apoptotic condensed nuclei). Intracellular ROS level was assessed by DHED-based fluorescent staining (middle panel-red, thin arrow marks ROS in non-apoptotic cells, thick arrow marks ROS in apoptotic cells), and merged figure (left) to show that ROS levels in non-apoptotic cells are lower than that in apoptotic cells (white boxes marked areas were enlarged (Zoom in)). **B**, Huh7 cells were treated with DMSO (Con), Lexa (1 µg/ml), CHX (10 µg/ml), NAC (10 mM), a combination of Lexa (1 µg/ml) and CHX (10 µg/ml) in the absence or presence of NAC (10 mM). Intracellular ROS level was measured with DHED dye (red fluorescence). **C**, Huh7 cells were treated with various stimuli as indicated and apoptosis was assessed by DNA ladder. **D**, Huh7 cells treated with DMSO (Con), Lexa (1 µg/ml), CHX (10 µg/ml), NAC (10 mM), the combination of Lexa (1 µg/ml) and CHX (10 µg/ml) in the absence or the presence of NAC (10 µM) for 6 h. Then cells were stained with Hoechst 33258 to test apoptotic cell death (representative apoptotic cells are marked with arrows). **E** and **F**, Huh7 cells were treated with conditions as indicated. Apoptosis was evaluated as in Fig. 1C and 1D. Data represent the mean values of three independent experiments (**p<0.05).

To assess whether ROS plays a major role in regulating Lexa and CHX combination-induced apoptosis, HCC cells were treated with Lexa and CHX for 6 h. Intracellular ROS levels were determined by a DHED-based fluorescence assay. As shown in Fig. 3A, Lexa and CHX combination treatment induced ROS dramatic increase in apoptotic cells, but not in non-apoptotic cells, suggesting ROS may contribute to CHX-and Lexa-induced apoptosis. To further determine whether generation of ROS can regulate apoptosis, N-acetyl-L-cysteine (NAC), a precursor of reduced glutathione (GSH) widely used as a thiol-containing antioxidant to scavenge intracellular ROS, was added before administration of Lexa and CHX. As shown in Fig. 3B, the ROS increase induced by combined treatment of Lexa and CHX was completely negated. More importantly, apoptosis assays indicated that the blockade of ROS by NAC can also protect cells against Lexa-and CHX-induced apoptosis (Fig. 3C, 3D, 3E, and 3F). These results suggest that Lexa and CHX combination treatment-induced ROS increase can trigger apoptosis in HCC cells.

### Effects of combination treatment with Lexa and CHX on protein expression in HCC cells

**Figure 4:**
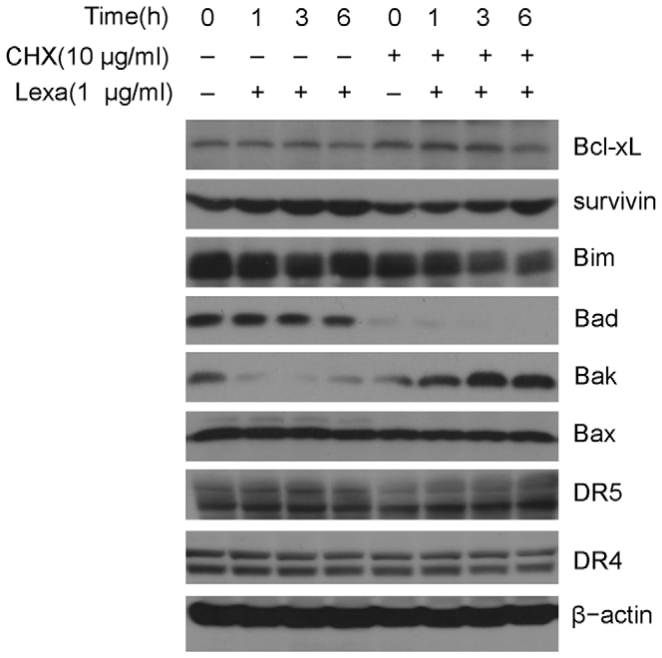
Protein expression in HCC cells treated with Lexa or the combination of Lexa and CHX. LH86 cells were treated with Lexa (1 µg/ml) or pre-treated with CHX (10 µg/ml) followed by Lexa (1 µg/ml) for up to 6 h. Cells were harvested and cell lysates were prepared for Western blotting. Bcl-xL, survivin, Bim, Bad, Bak, Bax, DR4, and DR5 were detected with specific antibodies respectively. β-actin was detected with anti-β-actin mouse antibody for an equal protein loading control.

Since CHX is a protein synthesis inhibitor, it may sensitize Lexa to induce apoptosis through down-regulating anti-apoptotic proteins. We analyzed the apoptosis associated protein expression changes in HCC cells treated with Lexa alone or combination-treated with Lexa and CHX for up to 6 h. Western blotting results showed that there were no decreases in anti-apoptotic molecules Bcl-xL or survivin expression; of the pro-apoptotic proteins, both Bad and Bim were down-regulated, while Bak was down-regulated with Lexa single treatment and up-regulated with combination treatment. Additionally, protein levels of pro-apoptotic Bax were not affected, and the expression levels of death receptors DR4 and DR5 showed no significant changes (Fig. 4). These results suggest that the inhibitor CHX could not completely block protein synthesis stimulated by Lexa in HCC cells and that apoptosis induced by combination treatment of Lexa and CHX may be mediated by Bax/Bak activation.

### Combination treatment of Lexa and CHX triggers the activation of Bax

**Figure 5:**
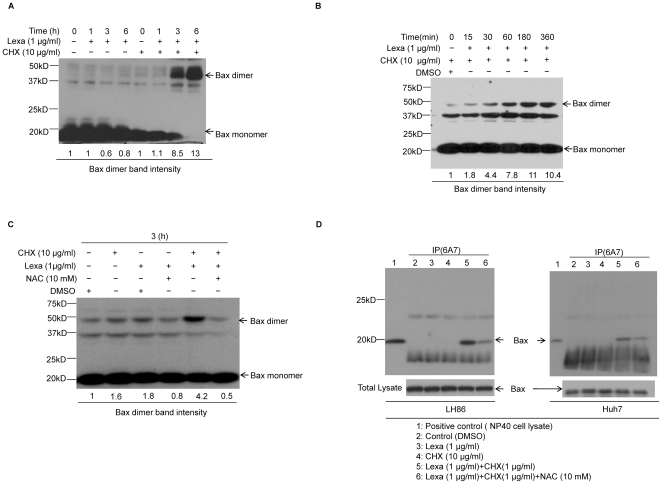
Lexa and CHX co-treatment-induced ROS increase triggers Bax activation. **A**, LH86 cells were treated with Lexa (1 µg/ml) or pre-treated with CHX (10 µg/ml) followed by Lexa (1 µg/ml) for up to 6 h. Cells were then treated with the cross-linking agent DSS. Bax proteins were resolved by SDS-PAGE and detected by Western blotting using a Bax polyclonal antibody. A 23 kDa band represents Bax monomers, and bands at 46 kDa, detected only in the presence of DSS, represent Bax homodimers. Relative intensity of Bax dimer bands was analyzed by Image J software. **B**, Huh7 cells were treated with a combination of Lexa (1 µg/ml) and CHX(10 µg/ml) for up to 6 h. Cell lysates were prepared and incubated with cross linker BMH as described in Materials and Methods. Cell lysates and cross linker mixture was boiled with 1×SDS loading buffer and Bax oligomerization was analyzed by Western blotting. The Bax dimer was recognized by Bax polyclonal antibody. Relative intensity of Bax dimer bands was quantified by Image J software. **C**, Huh7 cells treated with conditions as indicated and were harvested and extracts were prepared for Western blotting analysis to analyze Bax dimer formation. Relative intensity of Bax dimer bands was qualified by Image J software. D, LH86 (right panel) or Huh 7 (left panel) cells treated with various stimuli as indicated were harvested and lysed in Chaps lysis buffer and subjected to immunoprecipitation with anti-Bax 6A7 antibody for detection of conformationally changed Bax protein. Cell lysate obtained by NP-40 lysis was used as positive control. In addition, the total lysates were applied directly to SDS-PAGE/Western blotting analysis with specific anti-Bax polyclonal antibody and severed as loading control.

It is well known that under various cellular stresses, pro-apoptotic Bax/Bak can be activated through oligomerization and conformation change, resulting in mitochondria-dependent apoptosis [32]–[35]. Therefore, we examined whether Lexa and CHX co-treatment can activate Bax through oligomerization in HCC cells. Our results revealed that in the presence of CHX (10 µg/ml), Bax dimerization was up-regulated by Lexa in HCC cells (Fig. 5A, 5B, and 5C). To further test whether ROS was involved in Bax dimerization, cells were pre-incubated with NAC before the addition of Lexa and CHX. As shown in Fig. 5C, pre-treatment with NAC resulted in significant inhibition of Bax dimerization induced by combination treatment of Lexa and CHX.

Next we attempted to test the possibility that intracellular ROS could induce Bax to undergo conformation change in HCC cells combination-treated with Lexa and CHX. To this end, a Bax monoclonal antibody 6A7 that specifically recognizes conformationally changed Bax was used to do immunoprecipitation. As shown in Fig. 5D, combination treatment of Lexa and CHX could induce Bax conformation change in both LH86 and Huh7 HCC cells. As expected, with the addition of antioxidants NAC to block ROS, conformationally changed Bax was significant decreased.

**Figure 6:**
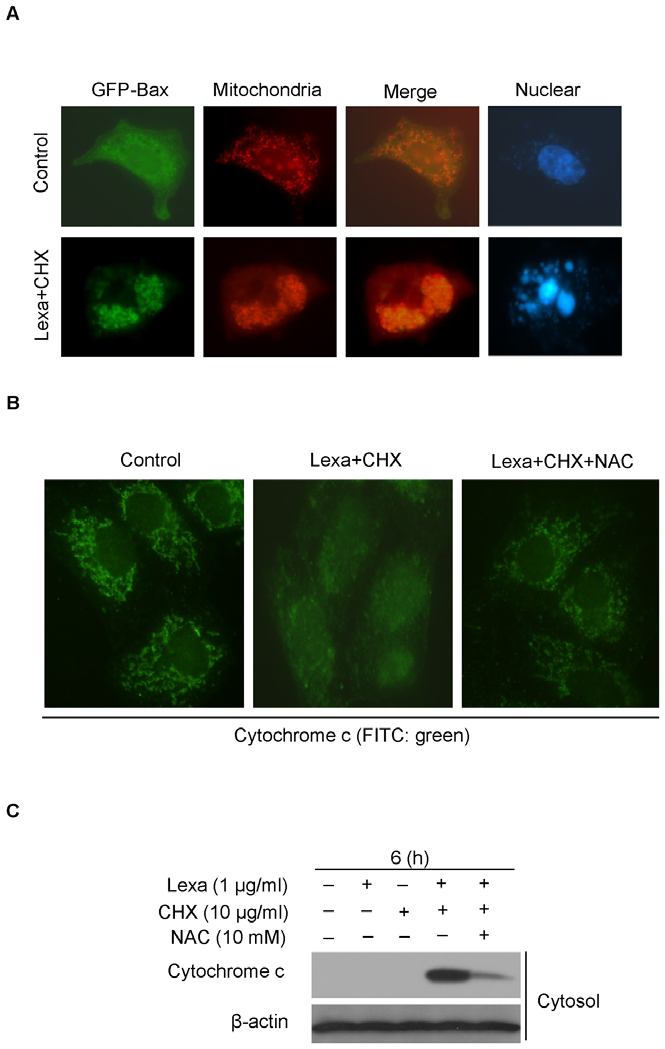
Lexa and CHX co-treatment induces Bax translocation and cytochrome c release in HCC cells. **A**, Huh7 cells were transfected with GFP-N1-Bax plasmid (0.25 µg/ml). After 10 h post-transfection, cells were treated with Lexa (1 µg/ml) in the presence of CHX (10 µg/ml) or not. GFP-N1-Bax translocation (green) was observed under fluorescent microscope. Mitochondria were stained with Mito Tracker (red) CMXRos (con, control; mito, mitochondria). Nuclear was stained with DAPI (blue). **B**, Huh7 cells cultured on cover slips were treated with Lexa (1 µg/ml) and CHX (10 µg/ml) in the presence (right panel) or absence (middle panel) of NAC (10 mM) for 6 h. Fluorescence immunostaining was performed to label cytochrome c with anti-cytochrome c primary antibody and FITC conjugated goat anti-mouse secondary antibody (green). **C**, Huh7 cells were exposed to different conditions as indicated and subjected to subcellular fractionation. Then, the cytosolic fractions were analyzed by Western blotting with antibody specific for cytochrome c. β-actin was detected with anti-β-actin mouse antibody for equal protein loading control.

After oligomerization and conformational change, the activated Bax can translocate to mitochondria, which results in cytochrome c release. In our study, we observed transiently transfected GFP-Bax translocation to mitochondria in cells treated with Lexa and CHX for 6 h (Fig. 6A). Next, we studied cytochrome c release from mitochondria in cells with combinational treatment of Lexa and CHX. Immunofluorescence staining results indicated that in cells treated with Lexa and CHX, cytochrome c was released and diffused into the cytosol (Fig. 6B, center), but this process was blocked by NAC (Fig. 6B, right panel). These observations were consistent with the results presented above: Lexa and CHX co-treatment-induced apoptosis can be negated by NAC (Fig. 3C, 3D, 3E, and 3F). Meanwhile, subcellular fractionation assay also showed Lexa and CHX combination treatment induced cytochrome c release in HCC cells, whereas, NAC addition significantly could block this process (Fig. 6C). Taken together, these results suggest that Lexa and CHX combination treatment-induced ROS generation results in apoptosis via the activation of Bax.

### HSP90 is involved in Lexa and CHX combination treatment-induced ROS and apoptosis

**Figure 7:**
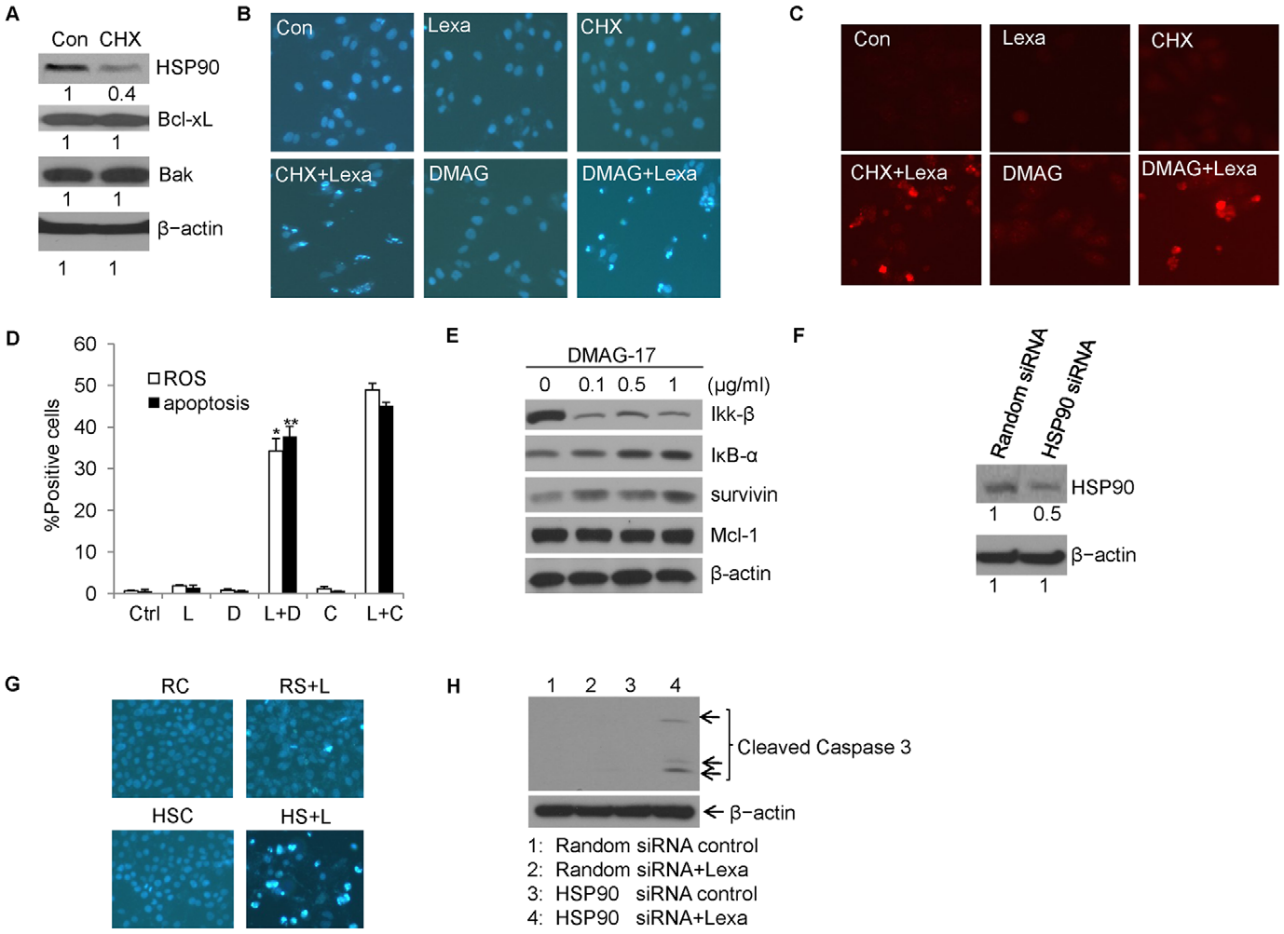
HSP90 is involved in Lexa and CHX co-treatment-induced ROS increase and apoptosis. **A**, Huh7 cells were treated with CHX (10 µg/ml) or not. Cell lysates were prepared for Western blotting. Anti-HSP90, anti-Bax, anti-Bcl-xL polyclonal antibodies were used to detect HSP90, Bax and Bcl-xL protein levels respectively. β-actin was used as an equal protein loading control. Relative intensities of bands in Fig. 7A were analyzed by Image J software. **B**, Huh7 cells were treated with DMSO (Con), Lexa (1 µg/ml), CHX (10 µg/ml), the combination of Lexa (1 µg/ml) and CHX (10 µg/ml), DMAG (0.5 µg/ml), or the combination of Lexa (1 µg/ml) and DMAG (0.5 µg/ml) for 6 h (Cells were treated with combination of Lexa and CHX as positive control). Apoptosis was assessed by nuclear morphological changes. **C**, Huh7 cells were treated as in Fig. 7B and intracellular ROS level was detected by fluorescent staining with DFHD (Cells were treated with combination of Lexa and CHX as positive control). **D**, Huh7 cells were treated with various conditions as indicated (Ctrl, control; L, Lexa (1 µg/ml); C, CHX (10 µg/ml), D, DMAG-17 (0.5 µg/ml); L+D, the combination of Lexa (1 µg/ml) and DMAG-17 (0.5 µg/ml); L+C, the combination of Lexa (1 µg/ml) and CHX (10 µg/ml). Cells with both ROS positive and DNA fragmentation were counted. Data represent the mean values of three independent experiments (*p<0.05; **p<0.05). **E**, Huh7 cells were treated with different dosage of DMAG-17 for 24 h. Cells were harvested and cell lysates were prepared for Western blotting. IKK-β, IκB-α, survivin and Mcl-1 protein levels were detected using specific antibodies respectively. β-actin was detected by anti-β-actin mouse antibody for an equal protein loading control. **F**, Huh7 cells were transfected with synthesized random siRNA (as control) and HSP90 specific siRNA, and after 48 h post-transfection cells were subjected to Western blotting analysis with HSP90 polyclonal antibody. β-actin was used as an equal protein loading control. The intensity of immunoblot bands Fig. 7F was analyzed by Image J software. **G**, Huh7 cells were transfected with synthesized random control siRNA and HSP90 siRNA, and 48 h post-transfection, cells were treated with Lexa or not and then subjected to Hoechst staining for apoptosis analysis (RC, random siRNA control; RS+L, random siRNA cells treated with Lexa (1 µg/ml); HSC, HSP90 siRNA control; HS+L, HSP90 siRNA cells treated with Lexa (1 µg/ml)). **H**, Huh7 cells treated as in Fig. 7G were harvested and subjected to Western blotting analysis with caspase 3 (for cleaved bands only) polyclonal antibody. β-actin was detected with anti-β-actin mouse antibody for an equal protein loading control.

Heat shock protein 90 (HSP90) may confer a survival advantage to tumor cells through association with several regulators of apoptosis [36]–[38]. HSP90 binding to its client protein may protect against physiological oxidative stress [39], [40]. Here, our data demonstrated that CHX specifically decreased HSP90 protein expression (Fig. 7A). Then, we asked whether HSP90 plays a key role in regulating Lexa and CHX combination-induced apoptosis. Using DMAG-17, a specific HSP90 inhibitor to block its activity, we observed that HSP90 inhibition significantly enhanced Lexa treatment-induced apoptotic cell death (Fig. 7B and 7D) and ROS production (Fig. 7C and 7D) in HCC cells, suggesting HSP90 may be involved in Lexa-induced ROS generation. It has also been reported that DMAG-17 can induce apoptosis in tumor cells through down-regulating IKKs, Mcl-1, and survivin expression [41]. However, in this study, apoptosis assays indicated that HCC cells are insensitive to DMAG-17 single treatment (Fig.7B and 7D). Further Western blotting results showed that protein levels of IKK-β were decreased; IκB-α and the NF-κB targeted proteins survivin were increased; Mcl-1, another anti-apoptotic protein, was not affected by DMAG-17 treatment (Fig. 7E). Finally, to further confirm that HSP90 inactivation is critical for Lexa-induced apoptosis, HCC cells were transiently transfected with HSP90 specific siRNA to knockdown HSP90 expression and were treated with Lexa. Apoptosis assays indicated that HSP90 knockdown (Fig. 7F) significantly sensitized Lexa-induced apoptosis in HCC cells (Fig. 7G, and 7H). These results suggest that HSP90 can be targeted by CHX to regulate cell sensitivity to Lexa-induced apoptosis.

### Lexa and CHX combination treatment has no apoptotic effects on normal human hepatocytes

**Figure 8:**
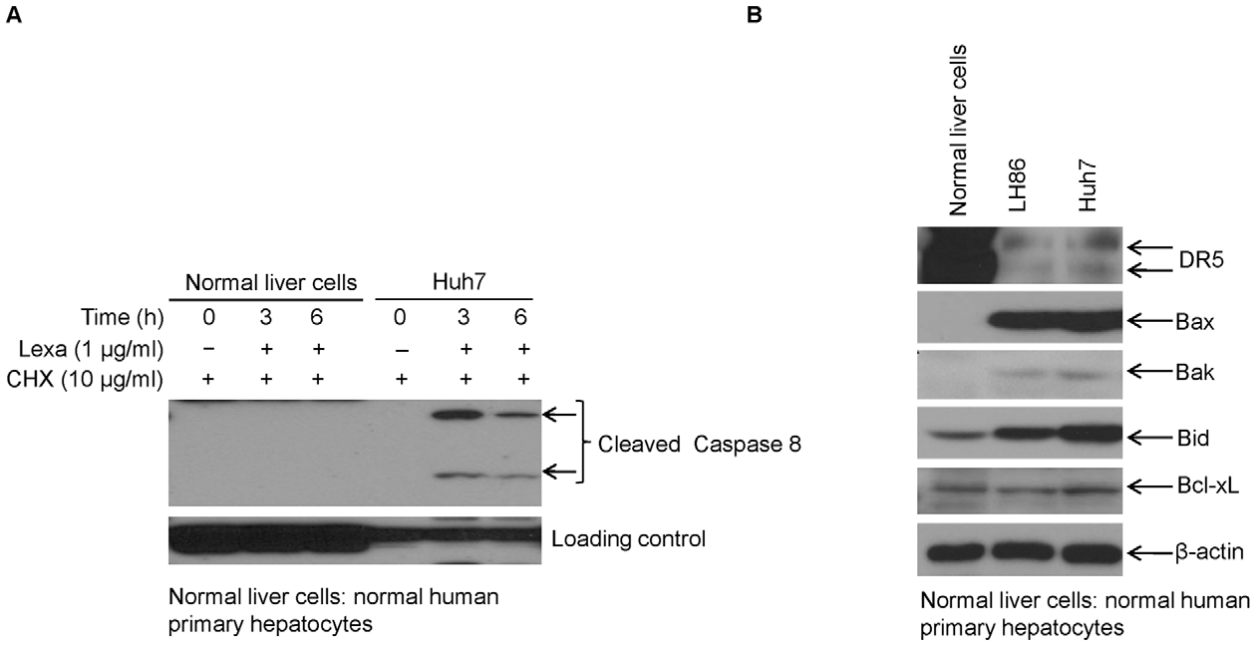
Lexa and CHX co-treatment has no lethal effects on normal human hepatocytes. **A**, Freshly prepared normal human primary hepatocytes were seeded on six-well plates and treated after two days with Lexa (1 µg/ml) and CHX (10 µg/ml) for up to 6 h. Huh7 cells treated under the same conditions were used as a positive control. Caspase 8 activation was measured through detecting the cleaved bands by Western blotting. **B**, Cell lysates from normal human hepatocytes and HCC cells LH86 and Huh7 were prepared and Western blotting was performed to detect Bcl-xL, Bax, Bak, Bim, Bid, and DR5 with specific antibodies.

A major barrier for the clinical use of TNF-α or TRAIL is their potential toxicity to normal human cells [42]–[45]. In this study, we examined whether Lexa and CHX combination treatment can selectively induce apoptosis in HCC cells. As shown in Fig. 8A, combination treatment with Lexa (1 µg/ml) and CHX (10 µg/ml) induced caspase 8 activation in HCC cells, but not in normal human hepatocytes. To further evaluate why normal hepatocytes are insensitive to CHX and Lexa co-treatment, we examined the expression of anti-apoptotic and pro-apoptotic proteins in HCC cells and normal human hepatocytes. In comparison with HCC cells, levels of pro-apoptotic Bax and Bak were undetectable in normal human hepatocytes, anti-apoptotic Bcl-xL levels were similar in all the cell lines tested, and Bid expression levels were much higher in HCC cells than those in normal human hepatocytes. However, DR5 protein in normal human hepatocytes showed much greater expression compared with that in HCC cells (Fig. 8B). These observations suggest that the insensitivity of human normal liver cells to Lexa and CHX combination treatment is not controlled by death receptor 5 (DR5) expression level on the cellular membrane, but rather determined by the expression levels of intracellular pro-apoptotic proteins, including Bax, Bak, and Bid. Taken together, these results suggest that CHX can selectively enhance Lexa to induce apoptosis in HCC cells.

## Discussion

In the present study, we investigated apoptosis induced by combination treatment with Lexa and CHX in resistant HCC cells. Our results demonstrated that pre-incubation with CHX could render resistant HCC cells sensitive to monoclonal antibody Lexa-induced apoptosis. Lexa and CHX combination treatment-induced apoptosis was triggered by intracellular ROS production. ROS accumulation in cells resulted in pro-apoptotic Bax/Bak activation through oligomerization and conformation change. HSP90 may act as a switch controlled by CHX to regulate intracellular ROS levels upon cell death stimulation. More interestingly, the combination treatment did not induce apoptosis in normal human hepatocytes comparing with the HCC cells under identical conditions. These findings are promising for the clinical use of CHX and Lexa as a novel chemotherapeutic option in the treatment of patients with HCC.

Lexa, a humanized monoclonal antibody, specifically binds to DR5 to activate death signals and has a longer half life *in vivo* rendering it as a potential killer of tumor cells. Here, we used HCC cells as a model to examine the apoptotic effects of Lexa. Our results showed that first, compared with positive control TNF-α, IκB-α phosphorylation could not be detected in HCC cells after treatment with Lexa, suggesting that specific binding to DR5 avoids activation of the NF-κB signaling pathway in HCC cells. This differs from the other two cytokines (TNF-α and TRAIL) that can induce activation of NF-κB to produce anti-apoptotic signals through binding to membrane [46]–[48]. Nevertheless, we did observe Lexa could reduce IκB-α expression in HCC cells, of which the significance remains to be clarified in the future. Second, single treatment of Lexa showed no lethal toxicity to HCC cells, which is consistent with previous studies reporting that HCC cells are resistant to TRAIL [49]–[51]. Third, we observed that pre-incubation with CHX could sensitize HCC cells to Lexa-induced apoptosis. It is therefore likely that besides inhibition of NF-κB, there are some other mechanisms through which CHX can sensitize Lexa to induce apoptosis in HCC cells.

Several groups have reported that activation of DR5 could signal non-apoptotic pathways through IKK, MAPK, P38, and JNK signaling pathways to neutralize TRAIL-induced apoptosis [52]–[54]. Our data demonstrated that specific inhibitors, including NBD, PD98059, SB203580, and SP600125, that respectively block the above mentioned anti-apoptotic signaling pathways, failed to reverse the resistance of HCC cells to apoptosis induced by Lexa, suggesting that CHX sensitizing cells to Lexa lethal toxicity does not require blocking anti-apoptotic effects from the activation of these signaling pathways.

As a pan-protein synthesis inhibitor, CHX may inhibit the activity of some apoptosis associated proteins by decreasing their expressions. However, our results demonstrated that CHX could not completely block protein synthesis because under CHX and Lexa co-treatment, Bak protein was significantly increased, suggesting that Bax/Bak may be activated to result in apoptotic cell death. As expected, our studies showed that Lexa and CHX co-treatment induced Bax oligomerization, conformational change, and translocation, which resulted in cytochrome c release and apoptosis. It has been reported that Bax/Bak can be activated by intracellular ROS to induce cell death in human colorectal cancer cells [55], [56]. Similarly in this study, when HCC cells were co-treated with CHX and Lexa, we observed ROS increase in apoptotic cells, but not in non-apoptotic ones. Moreover, the blockade of ROS with NAC not only prevented Bax oligomerization and conformational changes, but also decreased apoptosis significantly. These results suggest that generation of ROS plays a major role in CHX and Lexa co-treatment-induced apoptosis. Our findings are in agreement with a study that reported that the generation of ROS induced mitochondria-dependent apoptosis in colon cancer cells through Bax activation [57].

In comparison to treatment with either agent alone, Lexa-induced ROS accumulation occurred only in the presence of CHX, which suggests that CHX may modify some intracellular signaling pathway(s) or molecule(s) that act as a switch to control Lexa to induce ROS generation Our results based on this hypothesis indicated that pretreatment of cells with CHX decreased HSP90 expression significantly, and HSP90 inactivation by specific inhibitor DMAG-17 not only resulted in a Lexa-induced ROS increase, but also sensitized Lexa-induced apoptosis. Moreover, inhibition of HSP90 activity through gene silencing by specific siRNA significantly sensitized Lexa-induced apoptosis. These findings are also in agreement with a report showing that blockade of HSP90 sensitized prostate cancer cells to TRAIL [58]. Meanwhile, we have observed that DMAG-17 single treatment could not induce HCC cells to undergo apoptosis. Further analysis indicated that although IKK-β was decreased by DMAG-17, the NF-κB targeted proteins including survivin and Mcl-1 were not down-regulated. This is in contrast to a previous study which reported that DMAG-17 induced apoptosis in chronic lymphocytic leukemia (CLL) cells by decreasing NF-κB targeted gene transcription [59].

In order to bring the death receptor agonists closer to clinical therapy, we evaluated the safety of CHX and Lexa combination treatment in normal human hepatocytes. Our data indicated that normal human hepatocytes are resistant to this combination treatment. Comparison of DR5 expression levels and Lexa sensitivity of the normal hepatocytes and HCC cells used in this study did not reveal a consistent relationship, suggesting that Lexa sensitivity is not dependent on death receptor expression levels and that other intracellular mechanisms may control death signal transduction in resistant cells. As death receptor-mediated cell death can be regulated by Bcl-2 family BH3 only proteins, such as Bax, Bak, and Bid. Here, we found that expression levels of the BH3 only proteins Bax and Bak were undetectable in normal human hepatocytes, while the HCC cells showed clear high expression of these proteins. This obvious difference may be a key element in protecting normal cells against the toxicity of Lexa and CHX combination treatment. In addition, to extend the use of Lexa to treat HCC, we tested the effects of combination of the death receptor agonists with other chemotherapeutic reagents such as cisplatin or doxorubicin. Our data showed that only doxorubicin could sensitize Lexa-induced apoptosis in HCC cells (see Figure S1 and Figure S2). The underlying mechanism remains to be investigated.

In summary, we have found that HCC cells are generally resistant to Lexa, and CHX can sensitize HCC cells to Lexa-induced apoptosis. The underlying mechanism of action for combination treatment is related to ROS generation and Bax activation. Our study also demonstrates that normal hepatocytes are resistant to the combination therapy. We suggest that administration of CHX followed by Lexa should be considered for further investigation for the treatment of patients with HCC.

## Supporting Information

Figure S1 **Cisplatin (Cis) could not sensitize Lexatumumab (Lexa)-induced apoptosis in HCC cells.** Huh7 cells were treated with DMSO (control), Lexa (1 µg/ml), different doses of cisplatin (1-20 μg/ml), or a combination of Lexa and cisplatin as indicated. Apoptosis was measured by nuclear dye Hoechst 33258 staining to label DNA fragmentation (nuclear morphological changes). Note that Lexa, cisplatin, or the combination treatment of Lexa and cisplatin had no apoptotic toxicity to HCC cells.
(TIF)

Figure S2 **Doxorubicin (Dox) sensitizes Lexatumumab (Lexa)-induced apoptosis in HCC cells.** Huh7 cells were treated with DMSO (control), Lexa (1 µg/ml), different doses of doxorubicin (0.25-2 μM), or a combination of Lexa and doxorubicin as indicated. Apoptosis was measured by nuclear dye Hoechst 33258 staining to label DNA fragmentation (nuclear morphological changes). Note that either Lexa or doxorubicin could not induce apoptosis in HCC cells. However, the combination treatment of Lexa and doxorubicin induced significant apoptosis in HCC cells.
(TIF)
