# Supplementary figures and images for: Reactive Oxygen Species Is Essential for Cycloheximide to Sensitize Lexatumumab-Induced Apoptosis in Hepatocellular Carcinoma Cells
Source: PLoS One. 2011 Feb 10;6(2):e16966. doi: 10.1371/journal.pone.0016966 (PMC3037406; doi:10.1371/journal.pone.0016966)

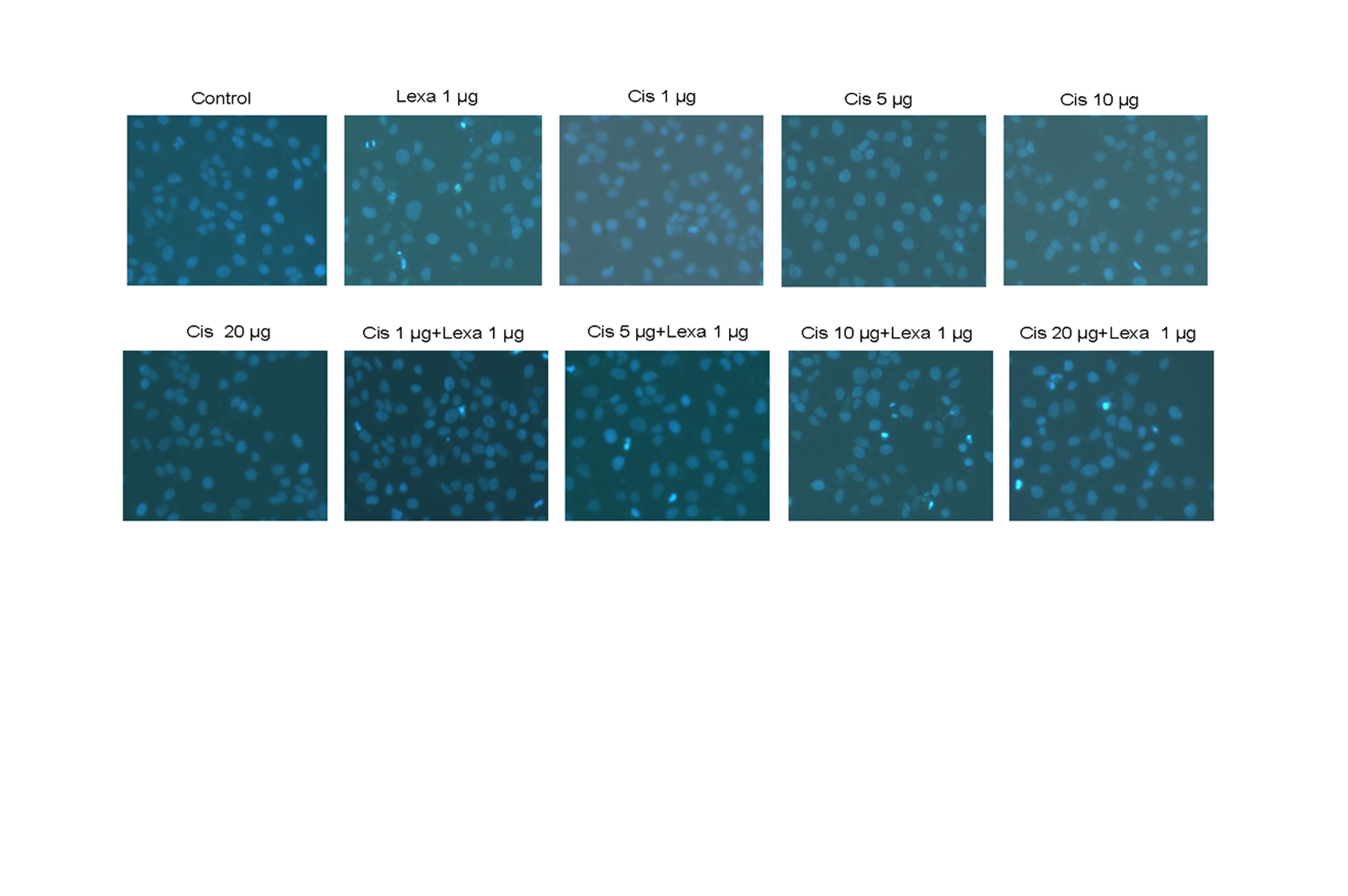

Supplement: Figure S1 — Cisplatin (Cis) could not sensitize Lexatumumab (Lexa)-induced apoptosis in HCC cells. Huh7 cells were treated with DMSO (control), Lexa (1 µg/ml), different doses of cisplatin (1-20 μg/ml), or a combination of Lexa and cisplatin as indicated. Apoptosis was measured by nuclear dye Hoechst 33258 staining to label DNA fragmentation (nuclear morphological changes). Note that Lexa, cisplatin, or the combination treatment of Lexa and cisplatin had no apoptotic toxicity to HCC cells. (TIF) [file pone.0016966.s001.tif]

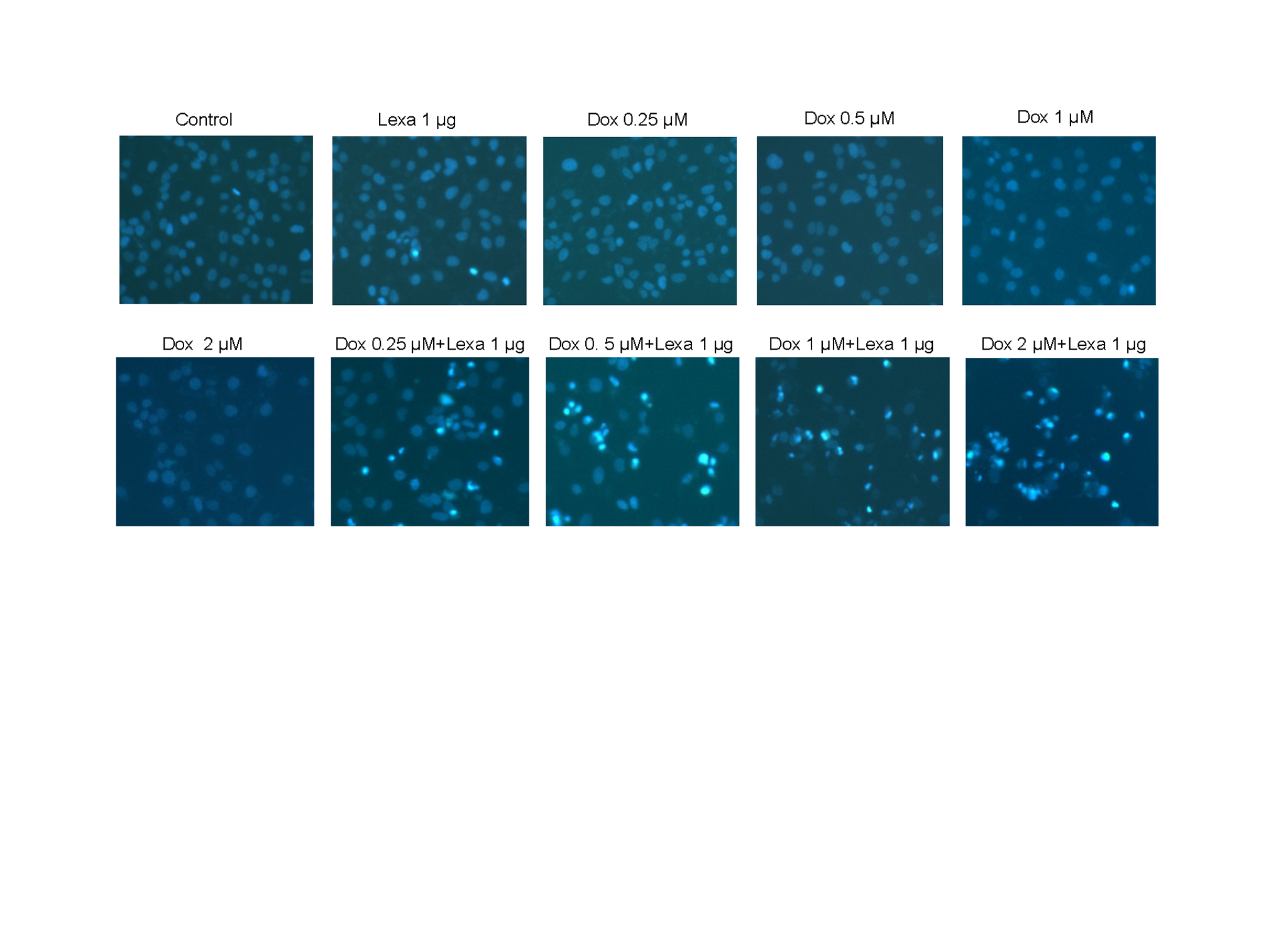

Supplement: Figure S2 — Doxorubicin (Dox) sensitizes Lexatumumab (Lexa)-induced apoptosis in HCC cells. Huh7 cells were treated with DMSO (control), Lexa (1 µg/ml), different doses of doxorubicin (0.25-2 μM), or a combination of Lexa and doxorubicin as indicated. Apoptosis was measured by nuclear dye Hoechst 33258 staining to label DNA fragmentation (nuclear morphological changes). Note that either Lexa or doxorubicin could not induce apoptosis in HCC cells. However, the combination treatment of Lexa and doxorubicin induced significant apoptosis in HCC cells. (TIF) [file pone.0016966.s002.tif]
